# Metabolic Regulation of Myeloid-Derived Suppressor Cell Function in Cancer

**DOI:** 10.3390/cells9041011

**Published:** 2020-04-18

**Authors:** Yufei Wang, Anna Jia, Yujing Bi, Yuexin Wang, Guangwei Liu

**Affiliations:** 1 Key Laboratory of Cell Proliferation and Regulation Biology, Ministry of Education, Institute of Cell Biology, College of Life Sciences, Beijing Normal University, Beijing 100875, China; 201821200044@mail.bnu.edu.cn (Y.W.); 201831200019@mail.bnu.edu.cn (A.J.); 201931200019@mail.bnu.edu.cn (Y.W.); 2 State Key Laboratory of Pathogen and Biosecurity, Beijing Institute of Microbiology and Epidemiology, Beijing 100071, China; byj7801@sina.com

**Keywords:** cancer, myeloid-derived suppressor cells, MDSCs, metabolism signaling pathway, immune cell differentiation, tumor immunotherapy, immunotherapy, cell differentiation

## Abstract

Myeloid-derived suppressor cells (MDSCs) are a group of immunosuppressive cells that play crucial roles in promoting tumor growth and protecting tumors from immune recognition in tumor-bearing mice and cancer patients. Recently, it has been shown that the metabolic activity of MDSCs plays an important role in the regulation of their inhibitory function, especially in the processes of tumor occurrence and development. The MDSC metabolism, such as glycolysis, fatty acid oxidation and amino acid metabolism, is rewired in the tumor microenvironment (TME), which enhances the immunosuppressive activity, resulting in effector T cell apoptosis and suppressive cell proliferation. Herein, we summarized the recent progress in the metabolic reprogramming and immunosuppressive function of MDSCs during tumorigenesis.

## 1. Introduction

Myeloid-derived suppressive cells (MDSCs), which were first discovered in the 1970s and finally identified and named in 2007, are a group of heterogeneous cells expanded through pathological activation from bone marrow-derived immature myeloid cells (IMCs) during autoimmune diseases, infections, cancer and graft vs. host disease (GVHD) [1,2,3]. Some studies have demonstrated the immunosuppressive function of MDSCs. Due to the negative regulatory activity of MDSCs, they play crucial roles in immune-associated diseases [4]. Especially in tumors, MDSCs discourage the antitumor response by interacting with other immune cells and modifying multiple signaling pathways, thereby accelerating tumor growth, expansion and immune escape, further leading to poor clinical outcomes [5,6]. Recently, intense efforts have focused on metabolic regulation, which is also important for MDSC enhancement of immunosuppressive activity, especially in cancer [7].

**Figure 1:**
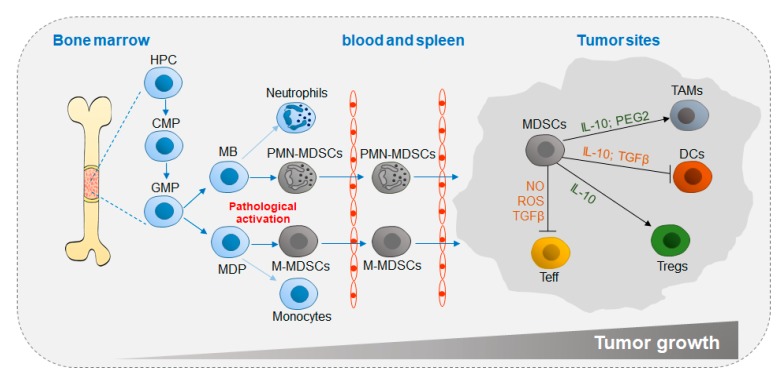
Differentiation and accumulation of MDSCs in the TME. Chronic inflammatory factors, such as G-CSF and GM-CSF, are secreted to promote myelopoiesis. Instead of neutrophils and monocytes, MDSCs originate from common myeloid progenitor cells under pathological conditions and migrate through the circulatory system to the tumor site, in which MDSCs exert immunosuppressive functions by generating anti-inflammatory cytokines. TME, tumor microenvironment; HPC, hemopoietic progenitor cell; CMP, common myeloid progenitor; GMP, granulocyte-macrophage progenitor; MB, myeloblast; MDP, monocyte/macrophage and dendritic cell precursor; MDSC, myeloid-derived suppressor cell; TAM, tumor-associated macrophage; DC, dendritic cell; T_reg_, regulatory T cell; T_eff_, effector T cell; IL-10, interleukin-10; PGE2, prostaglandin E2; TGFβ, transforming growth factor beta; IFNγ, interferon gamma; NO, nitric oxide; ROS, reactive oxygen species.

MDSC differentiation is closely related to tumor growth (Figure 1). In the tumor microenvironment (TME; pathological activation) in vivo, stimulation with tumor-derived factors (TDFs), such as vascular endothelial growth factor (VEGF) and granulocyte-macrophage colony-stimulating factors (GM-CSFs) induces MDSC differentiation in bone marrow (BM) from hemopoietic progenitor cells (HPCs) through common myeloid progenitors (CMPs) and granulocyte-macrophage progenitors (GMPs). Then, MDSCs circulate in the blood and spleen and eventually home to tumor sites, in which factors such as interleukin (IL-10) and transforming growth factor beta (TGFβ) secreted by MDSCs accelerate tumor growth by impeding antitumor activity and promoting suppressive cell differentiation [8,9].

In addition, MDSCs also contribute to metastases. Tumor metastasis is the process by which tumors invade from a primary site to other organs at a distance. The role of MDSCs in tumor metastasis mainly includes the following progressions: (1) remodeling the tumor microenvironment, reducing the antitumor immune response by suppressing T cells and natural killer (NK) cells, promoting the generation of immunosuppressive cells, such as regulatory T cells (T_reg_s) and regulatory B cells (B_reg_s) and promoting primary tumor growth; (2) promoting tumor epithelial-mesenchymal transition (EMT) and enabling tumors to acquire increased migration and invasion capabilities; (3) assisting tumor invasion of the blood stream and lymphatic vessels for migration; (4) establishing a premetastatic niche (pMN) for cancer cells implantation; (5) inducing tumor mesenchymal epithelial transition for expansion; and (6) promoting angiogenesis [10,11,12,13].

MDSCs are classified according to their surface marks. Based on phenotypic similarities to neutrophils and monocytes, murine MDSCs are divided into two major groups, monocytic MDSCs (M-MDSCs) and polymorphonuclear MDSCs (PMN-MDSCs) [9]. M-MDSCs are defined as CD11b^+^Ly6G^−It^Ly6C^hi^, and PMN-MDSCs are defined as CD11b^+^Ly6G^hi^Ly6C^lo^ [14,15]. There are three MDSC subsets in humans: M-MDSCs, PMN-MDSCs and early MDSCs (e-MDSCs). Among them, M-MDSCs are defined as CD11b^+^CD14^+^CD15^−^CD33^+^ HLA-DR^−^, PMN-MDSCs are defined as CD11b^+^CD14^−^CD15^+^ (or CD66b^+^) CD33^+^LOX-1^+^, and e-MDSCs are defined as Lin^−^HLA^−^DR^−^CD33^+^, where Lin includes CD3, CD14, CD15, CD19 and CD56 (Figure 1 and Table 1 and Table 2) [16,17,18].

## 2. Signaling Pathways that Regulate MDSC Functions

**Figure 2:**
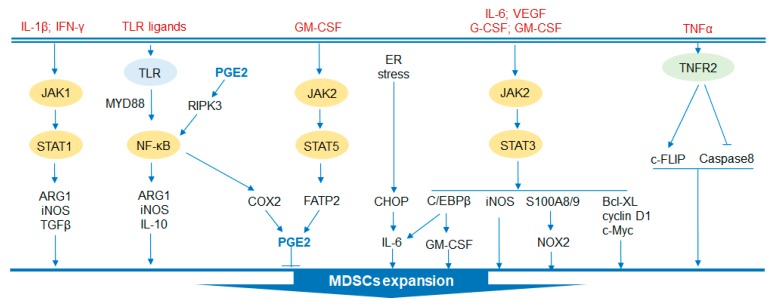
Regulatory signaling pathways in MDSC development and functions. Several signaling pathways are involved in the expansion of MDSCs. JAK2/STAT3 signaling enhances the immunosuppressive function of MDSCs by activating S100A8/9/NOX2 and iNOS to promote the generation of ROS. The pathway also protects MDSCs from apoptosis by expressing Bcl-XL, cyclin D1 and c-Myc. Moreover, this pathway promotes the activation of C/EBPβ. In addition, JAK1/STAT1 signaling accelerates the expansion of MDSCs by inducing the expression of ARG1, iNOS and TGFβ. Similarly, the proliferation of MDSCs can be accelerated by increased production of PGE2 through JAK2/STAT5 signaling. Furthermore, the TLR family also regulates the activation of MDSCs by activating NK-κB to generate protumor cytokines. CHOP is activated by ER stress and is involved in the activation of MDSCs. Additionally, TNFα-TNFR2 signaling is crucial to MDSC expansion by c-FLIP upregulation and caspase 8 reduction. JAK, Janus activated kinase; STAT, signal transducer and activator of transcription; ARG1, arginase 1; TLR, toll-like receptor; MyD88, myeloid differentiation factor 88; NF-κB, nuclear factor kappa-B; RIPK3, receptor-interacting protein kinase 3; COX2, cyclooxygenase-2; GM-CSF, granulocyte-macrophage colony stimulating factor; G-CSF, granulocyte-colony stimulating factor; NOX2, NADPH oxidase 2; iNOS, inducible nitric oxide synthase; FATP2, fatty acid transport protein 2; CHOP, C/EBP homologous protein; ER, endoplasmic reticulum; VEGF, vascular endothelial growth factor; Bcl-XL, B-cell lymphoma-XL; TNFα, tumor necrosis factor alpha; TNFR2, tumor necrosis factor receptor 2; c-FLIP, cellular FLICE (FADD-like IL1β-converting enzyme)-inhibitory protein.

The regulatory signaling pathways of MDSCs are now well established, as summarized in Figure 2. Regulation of the development and function of MDSCs is a complex process in which several signaling pathways are involved.

### 2.1. STAT Signaling Pathway

Signal transducer and activator of transcription (STAT), phosphorylated by the Janus activated kinase (JAK) family, is considered to play a critical role in the expansion of MDSCs [19]. STAT3, which has been extensively studied, prevents the apoptosis of MDSCs and promotes the expansion of MDSCs by mediating the expression of apoptosis inhibitors, including Bcl-XL, cyclin D and c-Myc [20,21,22]. In addition, activation of STAT3 drives the production of the calcium-binding protein S100A8/9, which is an inflammatory protein, and increases the accumulation of MDSCs by limiting dendritic cell (DC) differentiation [23]. Arginase 1 (ARG1) is also a downstream target of STAT3 in circulating and infiltrating MDSCs [24]. Similarly, NADPH oxidase 2 (NOX2), induced by STAT3 in MDSCs, generates reactive oxygen species (ROS) that can prevent DC differentiation [25]. Interleukin (IL)-1β and interferon gamma (IFNγ) activate STAT1 to produce ARG1 and inducible nitric oxide synthase (iNOS) and suppressive cytokines such as TGFβ to play a role in promoting tumor growth [26,27]. In contrast, a study showed that mice with STAT1 deficiency in breast cancer were more likely to have infiltration of PMN-MDSCs and tumor growth, which was rescued by anti-IL-17 treatment [28]. Therefore, STAT1 may have opposite effects on MDSCs under different conditions. In addition, a recent study indicated that STAT5, which is stimulated by GM-CSF, upregulates the expression of fatty acid transport protein 2 (FATP2) and exerts suppressive activity through the synthesis of prostaglandin E (PGE) [29].

### 2.2. C/EBPβ

CCAAT-enhancer-binding protein (C/EBP) β is a crucial regulator of myelopoiesis that is under the regulation of STAT3, which promotes HPC differentiation into MDSCs [30]. C/EBPβ deficiency significantly reduces iNOS and ARG1 in tumor-infiltrating MDSCs, and the suppressive activity of MDSCs is impaired [31]. C/EBP homologous protein (CHOP) is an apoptosis-related transcription factor that is induced by endoplasmic reticulum (ER) stress. CHOP is essential for the immunoregulatory function of MDSCs. CHOP-deficient MDSCs showed reduced immune-suppressive activity, mainly by inhibiting the C/EBPβ signaling pathway and thereby reducing the expression of IL-6 and phosphorylation of STAT3 [32]. MDSCs in mice express retinal noncoding RNA3 (RNCR3), which was upregulated by inflammatory and tumor-associated factors. RNCR3 was thought to promote the expression of CHOP by interacting with miR-185-5p [33]. miR-185-5p directly targets CHOP to affect the function and differentiation of MDSCs after knockdown of RNCR3 [33].

### 2.3. TLR Signaling Pathway

In the TME, the family of Toll-like receptors (TLRs) indirectly activates NF-κB through MyD88 to promote myelopoiesis via secreted IL-10, ARG1 and iNOS [34]. Signaling by TLR4 induces transformation of normal myeloid cells into functional MDSCs [35]. A study showed that activated TLR7/8 induces the transformation of M-MDSCs instead of PMN-MDSCs into antitumor M1-type macrophages. Conversely, activation of TLR1/2 induces M-MDSC differentiation into immunosuppressive M2-type macrophages [36]. Moreover, MDSCs that highly expressed TLR9 were detected in patients with prostate cancer and were accompanied by high levels of STAT3 and ARG1 [37]. Targeting MDSCs by unmethylated CpG oligodeoxynucleotide (CpG ODN, ligand for TLR9) effectively reduces the accumulation of MDSCs and increases tumor infiltrating cytotoxic T cells [37,38]. In addition, receptor-interacting protein kinase 3 (RIPK3) is an element that mediates programmed necrosis and is triggered by TLR3/TLR4 activation [39]. A reduction of RIPK3 in colorectal cancer is related to the accumulation of MDSCs. Decreased RIPK3 activates nuclear factor kappa-B (NF-κB) to transcribe the cyclooxygenase-2 (COX2) gene to generate PGE2, an inhibitor of RIPK3, further suppressing RIPK3 [40]. Moreover, tumor necrosis factor (TNFα) also contributes to the survival of MDSCs by interacting with tumor necrosis factor receptor 2 (TNFR2) to upregulate cellular FLICE (FADD-like IL1β-converting enzyme)-inhibitory protein (c-FLIP) and reduce expression of the protease caspase 8 [30,41].

## 3. Metabolic Reprogramming of MDSC Functions

**Figure 3:**
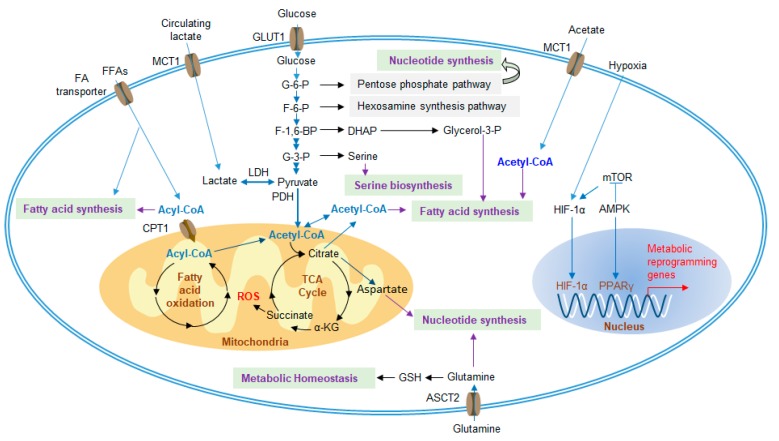
Metabolic regulatory pathways of MDSCs in the TME. Under hypoxic conditions, metabolic genes are upregulated, and increased metabolism enhances the suppressive function of MDSCs. MDSCs increase the uptake of extracellular nutrients, such as glucose, FA, glutamine and acetate, which are required for glycolysis, the TCA cycle, FAO, fatty acid synthesis and amino acid synthesis. Furthermore, excessive lactate, generated by tumor cells, can also be transported into MDSCs to participate in metabolism. FA, fatty acid; TCA cycle, tricarboxylic acid cycle; FAO, fatty acid oxidation; FFAs, free fatty acids; MCT1, monocarboxylate transporter 1; GLUT1, glucose transporter 1; G-6-P, glucose-6-phosphate; F-6-P, fructose-6-phosphate; F-1,6-P, fructose-1,6-bisphosphate; G-3-P, glyceraldehyde-3-phosphate; LDH, lactate dehydrogenase; PDH, pyruvate dehydrogenase; DHAP, dihydroxyacetone phosphate; CPT1, carnitine palmitoyltransferase 1; α-KG, alpha-ketoglutarate; mTOR, mammalian target of rapamycin; HIF-1α, hypoxia-inducible factor 1-alpha; AMPK, AMP-activated protein kinase; PPARγ, peroxisome proliferator-activator receptors gamma; GSH, glutathione; ASCT2, alanine-serine-cysteine transporter 2.

In the TME, changes in MDSC metabolic activity play important roles in the development and functional regulation of MDSCs. These metabolic signals mainly include glucose metabolism, lipid metabolism and amino acid metabolism (Figure 3).

### 3.1. Glycolysis and Lactate

Glycolysis is the main energy source of cells and can provide metabolites for nucleotide synthesis, hexosamine synthesis, amino acid synthesis, and fatty acid synthesis. In the 1920s, Otto Warburg was the one of the first to discover that metabolism could be reprogrammed to enhance glucose uptake and convert most of the pyruvate produced from glycolysis to lactate to exert an immunosuppressive effect in the TME, even if oxygen was abundant, which was named Warburg metabolism [42]. This characteristic metabolic reprogramming in tumors is a hallmark of cancer [43], which is caused by the high expression levels of tumor-related transcription factors, such as hypoxia-inducible factors 1-alpha (HIF-1α), c-Myc, and p53. These factors induce the expression of glucose transporters (GLUTs) to enhance glucose uptake and activate lactate dehydrogenase (LDH) to efficiently convert the produced pyruvate to lactate [44]. Excess lactate is transported across membranes by monocarboxylate transporters (MCT)1 and MCT4, which play vital roles in tumor aggressiveness [45,46]. Liver-enriched activator protein (LAP), an isoform of C/EBPβ, has been found in patients with triple-negative breast cancer and controls the expression of G-CSF and GM-CSF, thus promoting the development of MDSCs [47]. Restriction of aerobic glycolysis restrains the translation of LAP by stimulating AMPK-ULK and the autophagy pathway to impact the immunosuppression of MDSCs [47]. The glycolytic genes and metabolic rate of glycolysis in MDSCs are also upregulated in the TME to generate large amounts of phosphoenolpyruvate (PEP) by glycolysis, which, as an antioxidant agent, blunts the production of ROS to avoid ROS-mediated apoptosis [48]. In addition, the mammalian target of rapamycin (mTOR) positively regulates glycolysis in tumor-infiltrating M-MDSCs, accompanied by strong immunosuppressive activity, which can be counteracted by treatment with rapamycin (RPM) [49,50].

Lactate is considered an immunosuppressive metabolite that promotes tumor expansion, induces angiogenesis, stimulates amino acid metabolism, inhibits cytotoxic T cells, NK cells and DCs and further impedes the antitumor response [46]. In addition, lactate also promotes cancer growth by inducing protumor abilities, such as inducing MDSC differentiation [51]. The induced MDSCs in turn directly inhibit the antitumor response of immune cells [14,52]. Moreover, the number of immunosuppressive MDSCs increases when cultured with a high concentration of lactate [52].

### 3.2. Amino Acid Metabolism

Amino acids are essential nutrients for cell proliferation and immune recognition. In the TME, there are many ways to inhibit antitumor functions, including changes in amino acid metabolism. l-arginine (l-Arg) is a conditionally essential amino acid that is necessary for the activity of T lymphocytes. The catabolism of l-Arg is catalyzed by ARG1 or iNOS into urea and l-ornithine (Orn) or NO and l-citrulline [53]. MDSCs overexpress ARG1 under the stimulation of Th2 cytokines, such as IL-4, IL-10 and IL-13, while overexpress iNOS under the induction of Th1 cytokines, such as TNF-α, IL-1 and IFNγ [54]. MDSCs upregulate the expression of cationic amino acid transporter 2 (CAT2) in the TME, which increases the uptake of l-Arg [55]. l-Arg is depleted by MDSCs expressing ARG1, iNOS and CAT2, which impairs the T cell immune response [56]. Moreover, blocking CAT2 reverses the immunosuppressive activity of MDSCs [55]. l-Arg is a component of the T cell receptor (TCR) ζ chain. Depletion of l-ARG causes T cells to fail to recognize antigen and play an antitumor role [57,58]. PMN-MDSCs prefer ROS generation by activating STAT3 and NOX2. However, M-MDSCs augment the expression of iNOS to generate NO, stimulating apoptosis in T cells via impacts on STAT5 signaling. The reason why T cells undergo apoptosis is that L-Arg starvation, induced by iNOS and ARG1, causes T cells to stay in the G_0_-G_1_ phase of the cell cycle [13,54,59]. The depletion of amino acids can be sensed by general control nonderepressible 2 (GCN2) kinase. And GCN2 deficiency restores the proliferation of T cells [60]. The mTORC1 signaling pathway detects the reduction in amino acid metabolism and products, controlling cell cycle entry. In the TME, the depletion of l-ARG inhibits the mTORC1-mediated T cell antitumor response [61] In addition, MDSCs in mice or patients with autoimmunity increase the expression of ARG1 accompanied by Th17 differentiation, which has a proinflammatory phenotype. However, whether Th17 cells are involved in cancer remains unknown [62].

l-Cysteine (l-Cys) is an essential amino acid in mammals. MDSCs, with the overexpression of the transporter solute carrier family 7 member 11 (SLC7A11), can sequester cystine to reduce its concentration in the extracellular environment and convert it to l-Cys. In addition, l-Cys can also be synthesized from methionine catalyzed by cystathionase. Because of the lack of cystathionase and amino acid transporters in T cells [63], they can only be activated by taking up l-Cys delivered by DCs. Therefore, MDSCs effectively inhibit the antitumor function of T cells by sequestering l-cysteine [63,64].

l-Tryptophan (l-Trp) is also an essential amino acid that can be converted into kynurenine (Kyn), reducing the concentration of l-Trp in the TME by means of indoleamine 2,3-dioxygenase (IDO) and tryptophan-2,3-dioxygenase (TDO) and inhibiting the proliferation of T cells [65]. MDSCs overexpress IDO in response to inflammatory cytokines, such as IFNγ [66]. At the same time, studies have shown that Kyn may induce suppressive DCs and T_reg_s [67]. Furthermore, Kyn, catalyzed by IDO1, binds to the aryl hydrocarbon receptor (AhR) to activate downstream signaling and blunt anti-inflammatory activity [68].

### 3.3. Glutamine Metabolism

Glutamine is necessary to supply energy and substrates for tumor expansion, despite classification as a nonessential amino acid. Glutamine is the most abundant amino acid and is the material for nucleotide synthesis and de novo arginine synthesis [69]. Glutamine is also involved in the synthesis of glutathione (GSH), which maintains metabolic homeostasis. When malignant tumors occur, the uptake of glutamine is upregulated and mainly used for glutaminolysis. It is a process that glutamine is converted to glutamate, catalyzed in mitochondria by glutaminase, in both cancer cells and MDSCs. Then, glutamate is decomposed into alpha-ketoglutarate (α-KG), which is an intermediate of the TCA cycle, and further contributes to the synthesis of fatty acids, amino acids and GSH [7]. Culture with glutamine-limited medium impedes iNOS activity but not ARG1 in MDSCs [70]. Glutamine antagonism has been recently reported to significantly inhibit the metabolism of cancer cells and relevant signaling pathways. In contrast, effector T cells are observably activated through upregulation of oxidative metabolism and, as a result, enhance antitumor ability [71]. Taken together, glutaminolysis has been considered a therapeutic target by treating with glutamine antagonists, while the molecular mechanism by which cancer cells and T cells undergo metabolic plasticity and the effect on MDSCs remain unknown.

### 3.4. Lipid Metabolism

Altered lipid metabolism, which is relevant to hematopoietic activity and the risk of several immunological diseases, including cardiovascular disease, obesity and cancer, has recently received increasing attention [72,73]. Fatty acids provide an efficient way to generate energy via fatty acid oxidation (FAO, also known as β-oxidation), through which acetyl-CoA is produced to participate in the tricarboxylic acid (TCA) cycle, oxidative phosphorylation (OXPHOS) and fatty acid synthesis to meet the substantial cellular energy needs in the TME [74,75]. Several studies suggest that many immune cells undergo lipid metabolic reprogramming. For instance, the antitumor capacity of NK cells in obesity is blunted and fails to prevent tumor growth [76]. Recent studies have shown that the lipid metabolism of tumor-infiltrating MDSCs (T-MDSCs) is also transformed to increase fatty acid uptake and improve FAO, accompanied by an increase in mitochondrial mass, oxygen consumption rate (OCR) and expression of key FAO enzymes, including carnitine palmitoyltransferase 1 (CPT1), acyl CoA dehydrogenase (ACADM), peroxisome proliferator-activated receptor gamma coactivator 1-β (PGC1β), and 3-hydroxyacyl-CoA dehydrogenase (HADHA) [77].

Moreover, the upregulation of lipid metabolism also enhances the immunosuppressive functions of MDSCs. A study of the mechanism showed that T-MDSCs but not splenic MDSCs increase lipid uptake, which reveals that the fatty acid translocase CD36, induced by tumor-derived cytokines (G-CSF and GM-CSF) and targeted by the STAT3 and STAT5 signaling pathways, is relevant to FAO and immunosuppression of T-MDSCs [78]. Furthermore, fatty acid transport protein 2 (FATP2) is a long-chain fatty acid transporter that was reported to be overexpressed in mouse and human PMN-MDSCs but not M-MDSCs, is controlled via GM-CSF and STAT5, and exerts suppressive function by means of arachidonic acid uptake and synthesis of PGE2, which was blocked after FATP2 inhibition [29]. Liver X receptors (LXRs) are vital nuclear hormone receptor family transcription factors that participate in lipid homeostasis in mammals. Treatment with LXR agonists leads to apoptosis of MDSCs and a reduction in tumor volume, resulting from activated transcription target apolipoprotein E (ApoE), which binds with its receptor, expressed on MDSCs, and induces MDSC depletion, ultimately inhibiting tumor growth [79,80,81]. It has been reported that lectin-type oxidized LDL receptor 1 (LOX-1) is expressed on PMN-MDSCs of cancer patients but not healthy donors in response to endoplasmic reticulum (ER) stress, which seems to be a specific marker of human PMN-MDSCs [82].

In summary, these studies show that tumor-derived MDSCs are forced to reprogram lipid metabolism due to the massive accumulation of lipids and activation of related signaling pathways. Although inhibition of lipid metabolism can effectively limit tumor expansion, the molecular mechanism by which MDSCs increase fatty acid uptake and enhance their immunosuppressive effects still needs to be further explored.

### 3.5. Extracellular Adenosine

In addition, another molecule that is widely considered to play a significant role during tumorigenesis is extracellular adenosine (eADO). It accumulates under hypoxic conditions with dramatically high concentrations [83]. As a modulator, eADO blunts antitumor responses [84]. For instance, eADO reduces the activation of T cells by preventing the phosphorylation of related signaling molecules, such as Zap70, AKT and ERK1/2, and inhibits the expression of proinflammatory factors, including IFNγ, TNFα and perforin, on activated T cells [13,84,85]. Moreover, the development of MDSCs and T_reg_s is stimulated by adenosine to exert immunosuppressive effects [86].

eADO is generated from ATP through dephosphorylation by the ectoenzymes ectonucleoside triphosphate phosphohydrolase 1 (ENTPDase1/CD39) and ecto-5’-nucleotidase (Ecto5’NTase/CD73), which are highly expressed in MDSCs undergoing metabolic reprogramming [56]. The upregulation of these enzymes on MDSCs is induced by TGF-β-mTOR-HIF-1 signaling and has been reported in peripheral blood and tumor tissues from non-small cell lung cancer (NSCLC) patients [87]. Furthermore, treatment with metformin activates AMP-activated protein kinase α (AMPKα), which suppresses HIF-1 and downregulates the expression of CD39/CD73, impeding immunosuppression of MDSCs by enhancing the antitumor function of CD8^+^ T cells in patients with ovarian cancer [88].

Furthermore, eADO activates downstream signaling pathways by binding to G protein-coupled adenosine receptors, including A1R, A2AR, A2BR, and A3R, thereby generating an activating or inhibiting effect on different immune cells [89]. In particular, both A2AR and A2BR can affect most immune cells, such as NK cells, T cells, macrophages and MDSCs [90]. Many studies have revealed that A2AR, induced by adenosine, impedes the function and proliferation of T and NK cells; in contrast, A2AR stimulates Foxp3-expressing T_reg_s, which results in immune evasion of cancer and leads to poor clinical outcome [89,91,92]. Besides, it has been reported recently that A2BR is activated in response to HIF, suggesting that it is also involved in tumorigenesis. Indeed, A2BR plays a decisive role in the excitation of MDSCs and M2 macrophages [89,93,94].

Taken together, the increase in extracellular adenosine, as one of the major features of tumors, promotes a wide range of immunosuppressive functions. Therefore, reducing eADO-related signaling molecules could be a feasible therapeutic strategy.

## 4. Metabolic Activity of MDSCs in Cancer

### 4.1. Immunoregulatory Effects of MDSCs in Cancer

**Figure 4:**
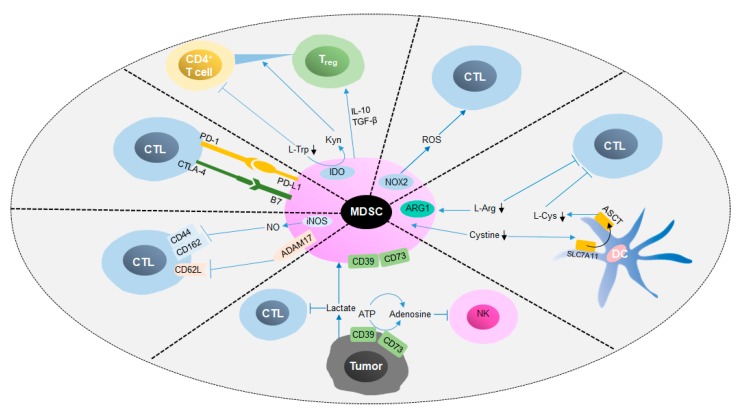
Immunosuppressive mechanisms of MDSCs in cancer. This figure shows that MDSCs exert immunosuppressive functions via cellular interactions and metabolic regulation. On the one hand, MDSCs inhibit antitumor immune cells, such as NK cells and CTLs, by inducing apoptosis and impacting homing. On the other hand, the differentiation of suppressive cells, such as T_reg_s, can be promoted by protumor cytokines secreted by MDSCs. CTL, cytotoxic T lymphocyte; NK cell, natural killer cell; PD-1, programmed death 1; PD-L1, programmed death ligand-1; CTLA-4, cytotoxic T lymphocyte-associated antigen 4; B7, costimulatory molecules; ADAM17, ADAM metallopeptidase domain 17; ATP, adenosine triphosphate; l-Arg, L-arginine; Kyn, kynurenine; IDO, indoleamine 2,3 dioxygenase; l-Cys, l-cysteine; l-Trp, L-tryptophan; SLC7A11, solute carrier family 7 member 11.

As mentioned above, MDSCs induce immune cell apoptosis and blunt anti-tumor responses by depleting amino acids, generating substantial amounts of ROS and reactive nitrogen species (RNS) and digesting ATP into ADO, thereby accelerate tumor growth (Figure 4) [56,84,88,95]. In addition, MDSCs inhibit the expression of l-selectin (CD62L) on T cells by binding with ADAM metallopeptidase domain 17 (ADAM17) on the T cell surface, resulting in an impact on lymphocyte homing [96,97]. Moreover, M-MDSCs isolated from spleen have been shown to cancel the induction of CD8^+^ cytotoxic T cells by CD62L, CD44, CD162 and granzyme B, which affects the binding of T cells with the extracellular matrix and selectins and leads to the suppression of T cells [98,99]. Programmed cell death-ligand 1 (PD-L1) and cytotoxic T-lymphocyte-associated protein 4 (CTLA-4) are widely identified immune checkpoints that negatively regulate the activation of T cells. Many studies have revealed that PD-L1, which is expressed on the membrane of MDSCs, interacts with programmed cell death-1 (PD-1) on T cells and induces the apoptosis of T cells [100]. Furthermore, production of Kyn and generation of IFNγ, IL-10 and TGF-β by MDSCs induces the differentiation of CD4^+^ naïve T cells to T_reg_s; however, the mechanism by which this occurs remains unclear [67,101,102]. Generally speaking, MDSCs inhibit the anti-tumor response and promote tumor development through metabolic regulation and the expression of surface molecules.

### 4.2. Metabolic Regulatory Mechanisms of MDSC Functions in Cancer

MDSCs have been shown to play key regulatory roles in the TME. Further studies have shown that the metabolic regulation of MDSCs is a critical mechanism in promoting tumor development and expansion. PMN-MDSCs (Table 3) and M-MDSCs (Table 4) play critical roles in many types of cancers through different metabolic mechanisms.

Lymphomas are malignant tumors derived from the lymphatic hematopoietic system and are divided into Hodgkin lymphoma (HL) and non-Hodgkin lymphoma (NHL) [106]. Several studies have uncovered that in NHL, including B cell NHL, diffuse large B cell lymphoma and NK/T cell NHL, increased expression of ARG1, IDO and iNOS in MDSCs is relevant to the enhancement of tumor growth and suppression of T cells [107,108,109,110]. However, little is known about whether the metabolic regulation of MDSCs participates in HL [111].

Lung cancer can be divided into small cell lung cancer (SCLC) and non-small cell lung cancer (NSCLC); SCLC is more malignant and has a lower survival rate than NSCLC [112]. In a mouse model, inhibition of FAO significantly decreases FA uptake and inhibits the function of MDSCs in the tumor site in Lewis lung carcinoma (3LL) [77]. In the same model, robust glycolysis activity is induced by mTOR activation in MDSCs [49]. Depletion of ARG1 diminishes the immunosuppressive function of MDSCs and restores the antitumor function of T cells in patients with NSCLC [113]. However, there are no studies on the metabolic regulation of MDSCs in SCLC.

In contrast to lung cancer and leukemia, altered metabolism of MDSCs also contributes to other cancers, such as melanoma, head and neck cancer (HNC) and colon cancer, in which enhanced glycolysis, lipid metabolism and increased expression of ARG1, IDO and iNOS are involved [77,82,105]. In summary, although many efforts have been made to reveal the mechanism by which MDSCs change the metabolism to promote tumor expansion, there are many questions to be answered.

## 5. The Therapeutic Effects of Targeting MDSCs

Pathologically activated MDSCs play central immunosuppressive roles in cancer. MDSCs change the nutritional system in the TME and interact with immune cells to inhibit antitumor activity, which leads to poor prognosis for cancer therapy. Therefore, many studies have been focused on developing therapeutic strategies. Current approaches targeting MDSCs mainly include elimination of MDSCs, suppression of immunosuppressive functions of MDSCs, promotion of MDSC differentiation into immune cells with no inhibitory activity, immune checkpoint treatment and inhibition of MDSC recruitment. The most direct targeted MDSC therapy is depletion of MDSCs. At present, there are several kinds of chemotherapy that can effectively eliminate MDSCs and enhance antitumor activity, mainly by low doses of 5-fluorouracil (5-FU), gemcitabine, cyclophosphamide, and paclitaxel [56,120]. Compared to gemcitabine, 5-FU is more effective and selective in reducing the number of MDSCs in the spleen and tumor sites of tumor-bearing mice [121]. However, 5-FU treatment stimulates assembly of the NOD-like receptor family pyrin domain-containing 3 (NLRP3) inflammasome in MDSCs, which increases the production of IL-1β and limits the therapeutic effect. Combined treatments with IL-1β inhibitors, such as docosahexaenoic acid (DHA), could be significant therapeutic approaches in tumor-bearing mice [121,122]. Moreover, the tyrosine kinase inhibitor sunitinib depletes MDSCs by inhibiting the interaction of c-kit, a factor needed for the accumulation of MDSCs, with its receptor [123]. VEGF is essential for MDSC expansion and tumorigenesis and is induced by phosphorylated STAT3, which can be inhibited by sunitinib [84]. Recently, liver-X nuclear receptor (LXR) activation by agonism RGX-104 has been shown to enhance the transcription of ApoE (genetic risk factor for Alzheimer’s disease), which combines with low density lipoprotein receptor-related protein 8 (LRP8) and induces the depletion of MDSCs [80].

Impeding the immunosuppressive function of MDSCs is a major cancer treatment strategy. PGE2 induces MDSCs to express ARG1, which plays a major immunosuppressive role, while COX2 is an upstream signal of PGE2 [40]. Silencing COX2 significantly reduces MDSCs in the spleens of tumor-bearing mice [124]. Inhibition of COX2 by inhibitors, such as acetylsalicylic acid or celecoxib, also significantly reduce the expression of ARG1, reduce the production of ROS, inhibit the function of MDSCs, and enhance antitumor activity [56,120]. Phosphodiesterase 5 (PDE5) is a hydrolase that specifically hydrolyzes cyclic guanosine monophosphate (cGMP), which induces MDSCs to produce ARG1 and iNOS to exert immunosuppressive activity. Studies show that inhibitors of PDE5 destroy the function of MDSCs and enhance the antitumor activity of T cells [56,120,125].

MDSCs can be induced to differentiate into mature myeloid cells, such as DCs and macrophages, reducing the level and immunosuppressive functions of MDSCs. All-trans retinoic acid (ATRA), a metabolite of vitamin A, has been well studied and induces MDSCs to differentiate into mature antigen-presenting cells (APCs) in vitro and in vivo, and reduce the restriction of T cells at the same time [126]. Treatment with ATRA significantly reduces MDSC levels in tumor-bearing mice and tumor patients [56,127]. The mechanism by which ATRA affects MDSCs is by upregulating the expression of glutathione through ERK1/2 activation to neutralize a large amount of ROS in MDSCs and promote MSDC differentiation [128].

Inhibiting MDSC migration could effectively reduce the level of MDSCs in the TME by preventing chemokines from binding to corresponding receptors on MDSCs. CXCR2 is a chemokine receptor on the surface of MDSCs. CXCR2 deficiency or CXCR2 inhibitors, such as SX-682 and AZD5069, eliminate MDSC metastasis and significantly enhance antitumor activity [129,130].

Tumors can suppress antitumor immunity through immune checkpoint molecules, including PD-L1, CTLA-4, and T cell immunoglobulin- and mucin-domain-containing molecule (Tim)-3. Therefore, there are a large number of immunotherapy drugs to treat tumors through targeted immune checkpoints, but only a few subsets of patients can be cured [131]. Many studies have shown that MDSCs are the main cause of poor outcome, and combination therapy can effectively cure cancer. The combination of SX-682 with anti-PD-1 has been reported to effectively enhance immune resistance [129,132]. Colony-stimulating factor 1 receptor (CSF-1R) is an important receptor for MDSC migration, and its inhibitor BLZ945 and anti-PD-1 combined with anti-PD-L1 can effectively cure cancer [133]. FATP2, which is overexpressed in PMN-MDSCs, improves cancer treatment efficacy by combination treatment with a FATP2 inhibitor and anti-CTLA-4 [29]. Several clinical trials are listed in Table 5.

## 6. Concluding Remarks

Generally, alterations in MDSC metabolism are a key driver of immunosuppression in a wide range of diseases, especially in cancer, and many studies have been conducted. However, there are still some important challenges to be faced at present, including how to further clarify the metabolic regulatory effects and detailed molecular regulatory mechanisms of MDSCs in cancer. Undoubtedly, the current metabolic regulatory mechanism of MDSCs provides important new ideas and research strategies for antitumor therapy targeting MDSCs.

## Figures and Tables

**Table 1:** Common molecules and functions of MDSCs in mice.

	M-MDSC	PMN-MDSC
	CD11b^+^Ly6G^−^Ly6C^hi^	CD11b^+^Ly6G^hi^Ly6C^lo^
Extracellular ROS	+	++
NO	++	−
ARG1	+	+
iNOS	+	−
PGE2	+	++
IL-10	+	+

**Table 2:** Common molecules and functions of MDSCs in humans.

	M-MDSC	PMN-MDSC	e-MDSC
	CD11b^+^CD14^+^CD15^−^CD33^+^ HLA-DR^−^	CD11b^+^CD14^+^ CD15^+^ (or CD66b^+^) CD33^+^LOX-1^+^	Lin^−^HLA-DR^−^CD33^+^
Extracellular ROS	+	++	+
NO	++	−	+
ARG1	+	++	+
iNOS	++	+	−
PGE2	+	++	N/A
IL-10	+	+	N/A

**Table 3:** PMN-MDSCs in cancer.

Pro-Tumor Targeting in MDSCs	Cancer Type	Metabolic Regulation Mechanism	Ref.
ARG1	Multiple myeloma	PMN-MDSCs are induced by multiple myeloma-related mesenchymal stem cells to have high expression of ARG1 and exert immunosuppressive function in tumor	[103]
	Multiple myeloma	PMN-MDSCs limit the anti-tumor response of T cells by increase the expression of ARG1 and other suppressive molecules, which is correlated with the expression of IL-18	[104]
	Head and neck cancer and urological cancers	Higher expression and activity of ARG1 in PMN-MDSCs, compared to M-MDSCs and e-MDSCs, contribute to potent pro-tumoral functions	[18]
CPT1	Renal cell carcinoma; Breast cancer; Colon cancer	PMN-MDSCs suppress immune response by increasing the expression of CPT1 and uptake of FA to promote FAO in tumors	[77]
mGluR2/3	Melanoma	PMN-MDSCs promote melanoma growth and inhibit proliferation of T cell via overexpressing metabotropic glutamate receptor (mGluR) 2/3	[105]
LOX-1	Non-small cell lung cancer (NSCLC); Head and neck cancer (HNC); Colon cancer	Lectin-type oxidized LDL receptor 1 (LOX-1), encoded at high levels in PMN-MDSCs, is related to ER stress and lipid metabolism in tumor	[82]
FATP2	EL4 lymphoma; Lewis lung carcinoma; CT26 colon carcinoma; Pancreatic cancer	Overexpression of fatty acid transport protein 2 (FATP2) in PMN-MDSCs is conductive to tumor growth by the synthesis of PGE2	[29]

**Table 4:** M-MDSCs in cancer.

Pro-Tumor Targeting in MDSCs	Cancer Type	Metabolic Regulation Mechanism	Ref.
mTOR	3LL Lewis lung carcinoma	Tumor-infiltrating M-MDSCs are associated with increased glycolysis induced by mTOR and display strong inhibitory abilities	[49]
iNOS	Non-small cell lung cancer	MDSCs with high expression of iNOS inhibit T cell functions, which leads to poor response to chemotherapy	[114]
	Ovarian cancer	Compared with healthy donors, the number of M-MDSCs increased in ovarian cancer, and the overexpression of iNOS was induced by STAT3	[115]
	Prostate cancer	High levels of iNOS overexpressing MDSCs are positive correlated with the number of T_reg_s	[116]
IDO	Gastric cancer	M-MDSCs (not PMN-MDSCs) produce IDO and blunt anti-tumor response of T cells	[117]
	Chronic lymphocytic leukemia	M-MDSCs suppress the activity of T cells and induce T_reg_s by increasing IDO activity	[118]
	Melanoma	IDO was highly expressed in M-MDSCs rather than PMN-MDSCs, and the IDO activity is positively correlated with tumor growth	[119]
CPT1	Renal cell carcinoma; Breast cancer; Colon cancer	M-MDSCs inhibit immune response by increasing the expression of CPT1 and uptake of FA to promote FAO in tumor	[77]
mGluR2/3	Melanoma	M-MDSCs promotes melanoma growth and inhibits the proliferation of T cells via metabotropic glutamate receptor (mGluR) 2/3	[105]

**Table 5:** Clinical trials targeting MDSCs.

	**Target**	**Agent**	**Combination Therapy**	**Cancer Type**	**Phase**	**Clinical Trial**
Depletion	MDSCs	Gemcitabine	Nivolumab	Non-small cell lung cancer	II	NCT03302247
		5-Fluorouracil	Bevacizumab	Glioblastoma	Recruiting	NCT02669173
		Cyclophosphamide/ Decitabine/Carboplatin/ Paclitaxel/Doxorubicin	Pembrolizumab	Breast cancer	II	NCT02957968
		RGX-104	Nivolumab	Advanced Solid Malignancies and Lymphoma	I	NCT02922764
		Ibrutinib	Nivolumab	Metastatic Malignant Solid Neoplasm	I	NCT03525925
Blocking recruitment and expansion	CCR5	Vicriviroc	Pembrolizumab	Advanced/metastatic microsatellite stable colorectal cancer	II	NCT03631407
		Leronlimab	Carboplatin	Metastatic Triple Negative Breast Cancer	I/II	NCT03838367
	CXCR2	SX-682	Pembrolizumab	Melanoma	Recruiting	NCT03161431
		AZD5069		Metastatic castration resistant prostate cancer	I	NCT03177187
	c-Kit	Imatinib		Chronic myeloid leukemia	II	NCT00852566
Inhibition of suppressive function	STAT3	AZD9150		Ovarian cancer and gastrointestinal cancer	II	NCT02417753
	COX2	Celecoxib	Cisplatin	Ovarian cancer	Recruiting	NCT02432378
	PDE5	Tadalafil		Head and Neck Squamous Cell Carcinoma	I	NCT02544880
	IDO	BMS-986205	Nivolumab	Glioblastoma	Recruiting	NCT04047706
	TLR7	Imiquimod	Paclitaxel	Breast cancer	II	NCT00821964
Promotion of differentiation	MDSCs	ATRA	Ipilimumab	Melanoma	II	NCT02403778
		ATRA	Pembrolizumab	Melanoma	Recruiting	NCT03200847
		Vitamin D		CLL		
Epigenetic therapy	HDAC	Entinostat	Nivolumab	Metastatic Cholangiocarcinoma and Pancreatic Cancer	Recruiting	NCT03250273
		Entinostat	Ipilimumab/ Nivolumab	Breast cancer	I	NCT02453620
